# Emerging Roles of Microglia in Blood-Brain Barrier Integrity in Aging and Neurodegeneration

**DOI:** 10.2174/1570159X21666230203103910

**Published:** 2024-09-01

**Authors:** Simeng Zhang, Rui Meng, Muzhou Jiang, Hong Qing, Junjun Ni

**Affiliations:** 1 Key Laboratory of Molecular Medicine and Biotherapy, Department of Biology, School of Life Science, Beijing Institute of Technology, Beijing, 100081, China;; 2 Liaoning Provincial Key Laboratory of Oral Diseases, Department of Periodontics, School and Hospital of Stomatology, China Medical University, Shenyang, 110002, China

**Keywords:** Microglia, neuroinflammation, blood-brain barrier, neurovascular unit, aging, neurodegeneration

## Abstract

The blood-brain barrier (BBB) is a highly selective interface between the blood and the brain parenchyma. It plays an essential role in maintaining a specialized environment for central nervous system function and homeostasis. The BBB disrupts with age, which contributes to the development of many age-related disorders due to central and peripheral toxic factors or BBB dysfunction. Microglia, the resident innate immune cells of the brain, have recently been explored for their ability to directly and indirectly regulate the integrity of the BBB. This review will focus on the current understanding of the molecular mechanisms utilized by microglia to regulate BBB integrity and how this becomes disrupted in aging and age-associated diseases. We will also discuss the rationale for considering microglia as a therapeutic target to prevent or slow down neurodegeneration.

## 1 INTRODUCTION

**Fig. (1):**
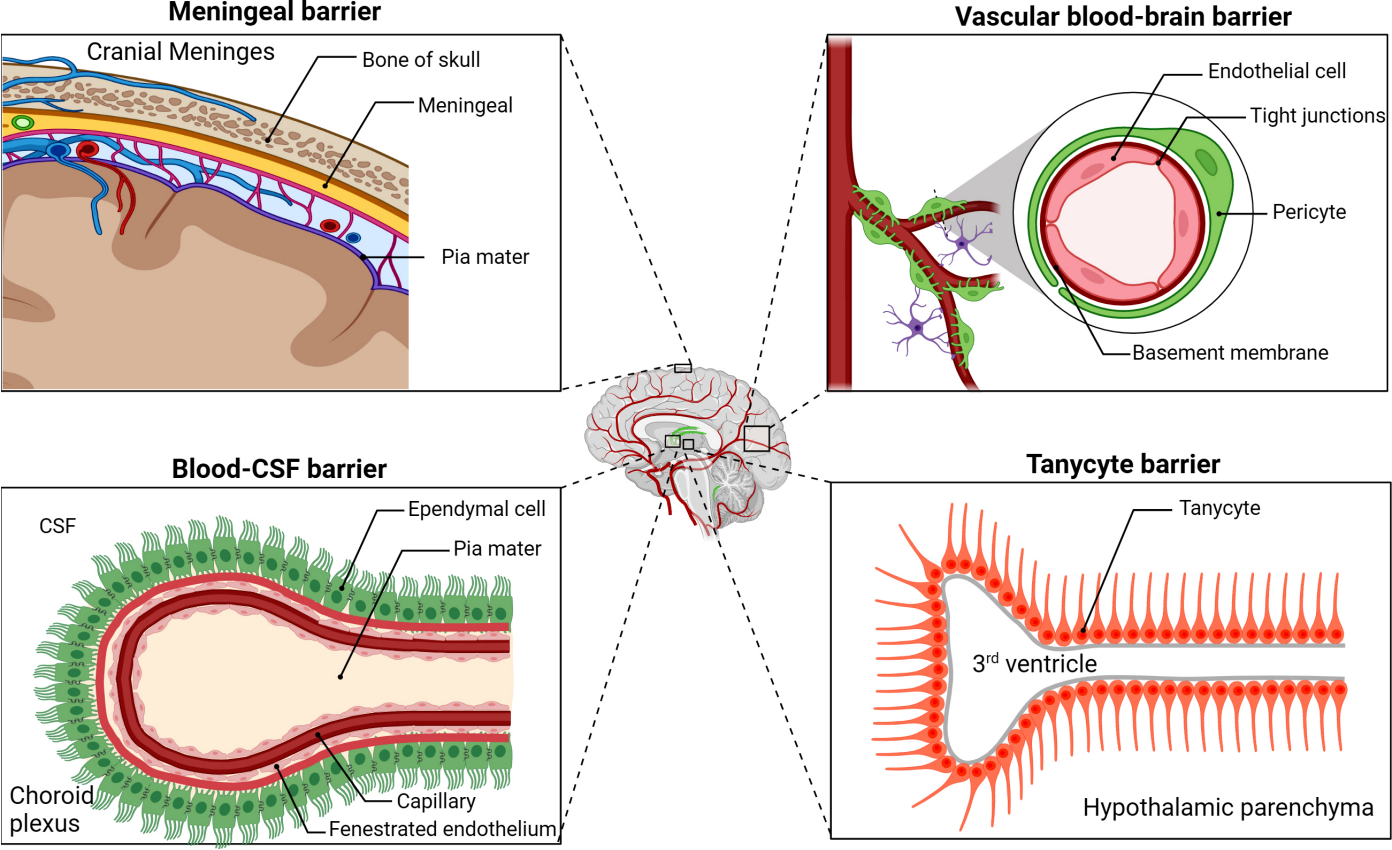
Schematic representation of the BBB barriers. The barriers of the BBB include the meningeal barrier, the vascular BBB, the blood-CSF barrier, and the tanycytic barrier. The meningeal barrier is found within the arachnoid matter, and is composed of epithelial cells. The vascular BBB is found at the capillary bed and the adjacent arterioles and venules and is composed of brain endothelial cells. The blood-CSF barrier resides at the choroid plexus and is composed of ependymal cells. The tanycytic barrier separates circumventricular organs from the adjacent area of the brain barrier.

Four different types of the blood-brain barrier (BBB) can be recognized based on the place where it is located: the vascular BBB (vBBB) is found in vertebrates at the capillary bed and the adjacent arterioles and venules; the blood-cerebrospinal fluid barrier is found in the choroid plexus; the tanycytic barrier separates circumventricular organs (small areas of the brain lacking a vBBB) from their adjacent area of the brain barrier; and finally, the meningeal barrier, which is primarily found within the arachnoid mater (Fig. **1**) [1]. Additionally, there are specialized extensions or regions of the BBB, such as the blood-retinal barrier and the otic barrier. By restricting the free diffusion of blood-borne molecules, the BBB establishes an isolated brain microenvironment by precisely balancing ionic concentrations needed for neural activity, compartmentalizes brain-specific growth factors and signaling molecules, and creates an immune-privileged brain environment [2].

Aging and age-associated diseases are associated with several alterations within the BBB, including focal necrosis of the cerebral endothelium, accumulation of extracellular matrix components in the vascular basement membrane, decreased endothelial mitochondrial density, and increased pinocytotic vessel wall thickness [3, 4]. Clinical evidence in humans suggests that BBB disruption in aging individuals is strongly correlated with cognitive decline and Alzheimer’s disease (AD) [5, 6]. A mechanistic understanding of the biological consequences of BBB breakdown would have the potential to reveal druggable targets for the intractable health problems of age-related disorders.

Microglia have been studied for decades. Due to a long history of experimental misinterpretation, their true origins remain debated. However, recent studies on microglial origin have suggested that they originate from erythromyeloid precursors in the yolk sac and subsequently invade the developing central nervous system (CNS) [7]. In the adult brain, microglia are normally sessile. However, they are also highly ramified, and continuously survey their environment with their long processes, constantly moving to identify any changes or the presence of pathogens [8]. Transcriptional profiling of microglia isolated from models of aging and different neurodegenerative diseases has revealed strikingly similar transcriptional networks. Karesman and colleagues have discovered disease-associated microglia (DAM), showing a unique transcriptional and functional signature *via* massively parallel single-cell RNA-seq analysis of CNS immune cells in neurodegenerative conditions [9]. The implication of crosstalk between microglia and other major brain cells, including neurons, astrocytes, and oligodendrocytes has been systemically reviewed [10-12]. The present review focuses on the emerging evidence supporting the role of microglia in the regulation of BBB integrity. Direct and indirect interactions of microglia with the BBB in aging and age-associated disorders have been discussed. We hope to illustrate the potential druggable targets by reviewing our current understanding of the mechanisms of microglial regulation on BBB integrity as well as recent progress in drug development.

## 2 THE BBB

Ehrlich first mentioned the existence of the BBB more than a hundred years ago [13]. The BBB is a special tissue, which is essential for the homeostasis of the cerebral microenvironment and normal neuronal functions. It is composed of endothelial cells, pericytes, astrocytes, neurons, and microglia, and together they are known as the neurovascular unit (NVU) [14]. It selectively allows substances to diffuse between the blood and brain to act as a line of defense *via* inter-endothelial adhesion, mediated by tight junctions (TJ) and adherens junctions (AJ) proteins [15]. The endothelial cells are surrounded by basement membranes, astrocytes, and pericytes [16]. Each component of BBB is responsible for maintaining barrier integrity.

There has been a great deal of focus on the molecular mechanism regulating the BBB, and it is closely associated with potential targets for the treatment of CNS disorders. In recent years, several imaging techniques have been developed to measure the integrity of the BBB *in vivo*, such as magnetic resonance imaging, computed tomography, single-photon emission computed tomography, or optical imaging. For example, real-time fluorescence imaging is now a common experimental method [14]. BBB dysfunction leads to capillary leakage, brain leukocyte infiltration, ingress of toxic substances, and upregulation of transforming growth factor-α (TGF-α) signaling in astrocytes, which disrupts the brain environment and leads to the loss of neurons [17]. A recent study has demonstrated that BBB breakdown could be considered a biomarker for the normal aging process [18]. Therefore, BBB integrity has critical pathophysiological implications.

### 2.1 Endothelial Cell

**Fig. (2):**
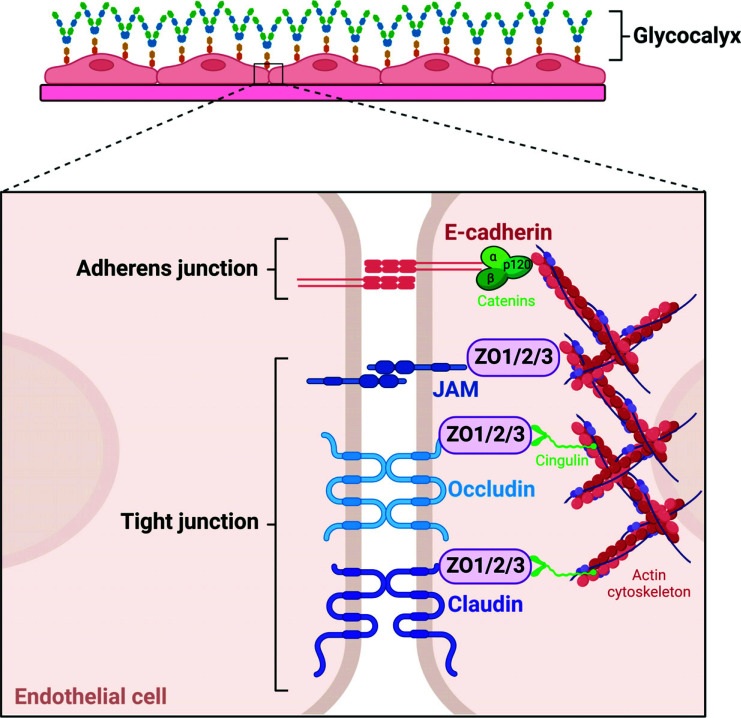
Schematic structures of the tight junctions (TJs) and adherens junctions (AJs). Major bicellular TJ proteins include three transmembrane proteins: occludins, claudins, and junctional adhesion molecules (JAMs), and cytoplasmatic zonula occludens proteins (ZO1, ZO2, and ZO3). Cadherin adhesion molecules are core AJ components, and all cadherins contain two or more extracellular cadherin domains. The cytoplasmic tail of cadherins binds to other cadherins. The ZOs and catenins interact with membrane proteins to anchor them to the actin cytoskeleton.

TJs and AJs between cerebral endothelial cells support the properties of BBB. They are recruited to the junctional complexes within lipid raft membrane microdomains and control the permeability of paracellular molecules across the BBB [15]. TJs maintain interendothelial continuity and vascular homeostasis, whereas AJs initiate and maintain endothelial cell-cell contacts (Fig. **2**) [19]. TJs form apical junctional complexes in specific endothelial cells. They include multiple proteins and are classified into two categories: integral membrane and cytoplasmic proteins.

Membrane proteins include occludins and claudins, which have N-and C-termini residing in the cytosol with two extracellular loop regions, and junctional adhesion molecules (JAMs). JAMs contain two IgG-like motifs with a single transmembrane domain. The cytoplasmic proteins, zonula occludens (ZO), are subdivided into ZO-1, ZO-2, and ZO-3. ZOs connect membrane proteins with actin cytoplasmic proteins *via* cingulin dimers providing the structural basis for tight junctions [20, 21]. The overexpression of occludins increases electrical resistance and reduces the permeability of molecule tracers [22].

Serine and threonine residues of occludins are phosphorylated, which is required for the assembly of TJs. Phosphorylation is regulated by balanced kinase and phosphatase activity and contributes to the permeability of the BBB. Protein kinase C (PKC) and tyrosine kinases can promote the hydrogen peroxide-induced disassembly of TJs and damage the interaction between occludins and ZO-1 [23].

Another main component of endothelial TJs is the claudin family. In particular, claudin-5 (Cldn5) can decrease paracellular iron movement and reduce paracellular clefts. Embryonic ablation of Cldn5 in mice leads to early postnatal brain oedema and lethality [24]. Cldn5 function is also modified by phosphorylation [25]. Rho kinase (RhoK), which belongs to the Rho family, is a regulatory molecule linking membrane receptors to the actin cytoskeleton. It can regulate the endothelial barrier and leukocyte migration by directly phosphorylating TJ proteins, including occludins and Cldn5 [23].

Other members of the claudin family also play a role in the BBB. Cadherin adhesion molecules are core AJ components. All cadherins contain two or more extracellular cadherin domains. Classic cadherins also have a highly conserved cytoplasmic tail that interacts with a defined set of cytoplasmic proteins, the catenins. p120 catenin and β-catenin bind to the cytoplasmic tail of cadherin, and β-catenin binds to α-catenin to form the cadherin-catenin complex (Fig. **2**) [26].

The endothelial glycocalyx (EG) is the main component of the vascular endothelial surface layer, which locates within the endothelial cell lumen to bind plasma proteins and soluble glycosaminoglycans. The EG contributes to the physical barrier between blood and endothelial cells, which includes vascular cell adhesion molecules (VCAM) and intercellular adhesion molecules (ICAM) tissue factor pathway inhibitors, nitric oxide synthase (NOS), and extracellular superoxide dismutase. In the state of disease, reduction of the EG can promote the adhesion between endothelial cells and leukocytes by exposing adhesion receptors. This leads to endothelium dysfunction and damage to the BBB [27, 28]. The EG carries a net negative charge, which can induce or interrupt the diffusion of proteins to the surface of endothelial cells. Moreover, it also protects BBB from oxidative stress [27].

### 2.2 Astrocytes

Basal astrocyte endfeet cover nearly the entire surface of the vascular endothelium in the brain and are involved in forming and maintaining the integrity of the BBB. As a result, astrocyte reactivity has been linked to CNS disorders [29]. The astrocyte endfeet contain the water channel, aquaporin 4 (AQP4), which is related to the migration of astrocytes and edema [30]. A recent study has demonstrated that damage of astrocytes can result in the transient destruction of the BBB and selective loss of occludins from TJs through using a model of AQP4-seropositive neuromyelitis optica spectrum disorder [31].

Astrocytes also release some cytokines and chemokines, such as C-C motif ligand 2 (CCL2) and C-X-C chemokine ligand 8 (CXCL8), and interact with various immune cells [32]. The polarization of astrocytes is similar to that of microglia and has two phenotypes. The A1 phenotype exhibits neurotoxic features and is associated with many neuroinflammatory diseases. The A2 phenotype is neuroprotective [33]. Astrocyte phenotypic shift is a novel regulator for maintaining normal BBB function. RNA sequencing showed that the BBB damage caused transcriptomic differences in the expression of several BBB regulatory genes in astrocytes, including proprotein convertase subtilisin/kexin type 1 inhibitor (Pcsk1n), protein phosphatase 1 regulatory inhibitor subunit 14A (Ppp1r14a), and complement fragment 4b (C4b) [34].

### 2.3 Pericytes

Pericytes interact with astrocytes, neurons, and endothelial cells, and play an important role in regulating and maintaining CNS homeostasis [35]. The ablation and dysfunction of pericytes are one of the hallmarks of BBB damage. In recent years, it has been confirmed that the loss of BBB structural integrity is caused by loss of pericytes and tight connections between astrocytes and endothelial cells *via* GFAP and PDGFR-β immunofluorescent staining in traumatic brain injury samples [36].

Pericytes, astrocytes, and endothelial cells are closely associated with the basement membrane, which is composed of four main proteins: collagen IV, laminin, nidogen, and perlecan. The basement membrane contributes to cell anchoring, structural support, and regulating immune cells and signaling pathways [37]. Pericytes and endothelial cells are co-embedded in the basement membrane by integrins, N-cadherin, gap junctional protein (connexin-43), and adherent junctional protein [38, 39]. They form direct synaptic-like peg-socket focal contacts with endothelium through N-cadherin and connexins, allowing an exchange of ions, metabolites, second messengers, and ribonucleic acids between the two cell types. N-cadherin is an adherent junctional protein that is expressed in brain endothelial cells and related to cerebral angiogenesis. It forms heterotypic adhesion between endothelial cells and surrounding vascular smooth muscle cells and pericytes. Connexin-43 is a transmembrane protein that can form gap junctions and is necessary for cytosolic proteins to diffuse [40]. A study illustrated that brain injury caused by fluid percussion injury is related to the dysfunction of pericytes and can lead to the reduction of integrins, N-cadherin, and connexin-43, downregulating the formation and expression of TJ proteins and increasing the expression of AQP4 [36]. These results reconfirm that the interaction of the components of the NVU contributes to BBB integrity.

## 3 INVOLVEMENT AND MEDIATION OF MICROGLIA IN INFLAMMATORY PROCESSES

### 3.1 The Expression of Microglial Markers

Microglia are resident immune cells whose progenitors arise from the peripheral mesodermal tissue [41]. They not only maintain neuronal health but also contribute to the development of neurons and the progression of neuroinflammation [42]. Microglia express specific macrophage markers on their cell surface, such as the macrophage colony-stimulating factor (M-CSF)-1 receptor (M-CSF-1R, CD115), the glycoproteins F4/80, the alpha-M integrin (CD11b), the fractalkine receptor (CX_3_CR1), the inhibitory immune receptor CD200R, the surface enzyme tyrosine-protein phosphatase nonreceptor-type substrate or CD172α, and the intracellular calcium-binding protein Iba-1 under physiological conditions [43].

Colony stimulating factor 1 receptor (CSF1R) is a receptor tyrosine kinase that is involved in the development and maintenance of microglia. Intracellular and extracellular signals are transmitted by CSF1R, which promotes microglia survival and proliferation [44]. CSF1R recognizes two ligands: CSF1 and interleukin (IL)-34. These are closely related to myeloid cell differentiation in the skin epidermis and CNS. The CSF1R inhibitor PLX3397 reduces the number of microglia in the adult brain. However, the depletion of microglia in this way does not affect the BBB, cognition, or behavior [45].

Although microglia are related to macrophages, they involve different functions and thus have specific markers. TMEM119 is a potent marker of microglia, which can differentiate resident microglia from blood-derived macrophages [46]. Further investigation into the role of microglia in the BBB will be useful for our understanding of CNS pathology.

### 3.2 Polarization and Regulation of Microglia

Activated microglia can be classified as M1 and M2 phenotypes, which have cytotoxic or neuroprotective roles in CNS disorders. The microglial phenotypic change depends on the signal microglia receive.

To respond to injury and infection, M1 microglia marked by CD16/32 and CD68 are the first line of defense, and facilitate the protective responses of clearing invading pathogens [47]. They also induce neurotoxicity triggering by toll-like receptors (TLR) and interferon (IFN)-γ signaling pathways and release various pro-inflammatory cytokines, such as tumor necrosis factor-α (TNF-α), IL-1β, superoxide, nitric oxide (NO), reactive oxygen species (ROS), inducible NOS (iNOS), IL-6, and proteases (Table **1**) [48].

Several published studies have reported the regulation and characteristics of these cytokines. For example, the morphology of microglia is distinct in transgenic mice with CNS-targeted chronic production of IL-6, with a reduction in microglial process length in the cerebellum and cortex, and the number of branching points, terminal points, and the total number of Sholl intersections. It also induces transcriptomic and molecular changes using principal component analysis on all genes that have passed the expression level criteria. Under glutamate receptor-mediated circumstances, both TNF-α and IL-1β have dose-dependent neurotoxic and neuroprotective effects [49]. However, the effect cannot be simultaneously toxic and protective. TNF-α and IL-1β are well-known pro-inflammatory cytokines and induce neuronal death *in vitro*; they can also increase both intracellular and extracellular glutamate levels [50].

M1 microglia activation is not always harmful to the neurons. It can also promote axonal regeneration in some specific cases. Similarly, the M2 microglial phenotype is not always beneficial. The mechanisms of microglial action in the pathophysiology of CNS disease are complex. A recent study has reconfirmed this concept by using P2X7, a ligand-gated ion channel receptor that elicits pro-inflammatory and pro-apoptotic actions, and further demonstrated the relevant signaling molecules in neuron-microglial crosstalk [51]. Two TNF-α receptor subtypes have opposite effects on AMPA-induced cell death [52]. Anti-inflammatory factors released by M2 microglia, such as IL-10 and TGF-β, could convert microglial phenotype from M1 to M2, which has become one of the particular focuses of microglia research [53].

Inhibition of CSF1R reduces neurological deficits and the expression of TNF-α. It also inhibits NLR family pyrin domain containing 3 (NLRP3) pathway activation, which is correlated with microglial activation. IL-4 promotes differentiation to M2 phenotype and reduces neuronal apoptosis in intracerebral hemorrhage (ICH) mice by alleviating the effects of inflammation following secondary brain injury [54]. However, the phenotype of microglia is not constant; it could be transformed under specific conditions. Quercetin is a natural polyphenol belonging to the flavonoid family, which is neuroprotective and reduces phagocytosis induced by lipopolysaccharides (LPS) [55]. Under quercetin treatment, NO and expression levels of the pro-inflammatory cytokines, iNOS, and cyclooxygenase (COX)-2 were also foound to be reduced [56]. COX-2 gene greatly responds to inflammatory factors, and IL-1β, TNF-α and LPS can induce an increase in COX-2 gene expression and the subsequent production of prostaglandins [57]. Additionally, the study demonstrated that quercetin reduces the expression of M1 markers and increases the expression of M2 markers, such as IL-10, and the endogenous antioxidants, heme oxygenase 1, glutamate-cysteine ligase catalytic subunit, glutamate-cysteine ligase modifier subunit, and NAD(P)H quinone oxidoreductase-1 (Table **1**) [56].

Anti-inflammatory therapies need to get access to CNS, but it is impractical to utilize cytokines that hardly cross the BBB to modulate neuroinflammation. Minocycline is one of the tetracycline antibiotics and a commonly useful tool to explore the mechanisms of microglial polarization. It can permeate into the CNS and selectively inhibit M1 polarization. Furthermore, triggering receptors expressed on myeloid cells 2 (TREM2) play a vital role in M2 polarization. Zhang and colleagues found that M1 microglial responses are exaggerated by using TREM2 knock-down mice. The overexpression of TREM2 leads to M2 microglia polarization and alleviation of microglial inflammation [58].

### 3.3 Involvement of Microglia in Regulating BBB

BBB restricts molecules that can reach the brain parenchyma, which is surrounded by vasculature with three layers, including endothelium, basal membranes, and extracellular matrix (ECM). Glia cells and the ECM have an important role in maintaining brain homeostasis. Matrix metalloproteinases (MMPs) are endopeptidases that are involved in neuronal plasticity and affect the ECM [59]. MMP transcription is regulated by pro-inflammatory cytokines, growth factors, and hormones [60]. IκB-α, an endogenous inhibitor of NF-κB, is phosphorylated and degraded after LPS treatment, followed by liberation and phosphorylation of NF-κB, thus resulting in transcriptional upregulation of inflammatory mediators [61]. LPS-induced phosphorylation of NF-κB and IκB-α could be reversed by ECM treatment. Therefore, the ECM could inhibit LPS-induced neuroinflammation and protect neurons from microglia-mediated inflammatory injury. Therefore, microglia and BBB have a close relationship, and the molecules involved in their homeostasis may be potential novel targets to treat neuroinflammation.

#### 3.3.1 The Dual Effects of Microglia on BBB

**Fig. (3):**
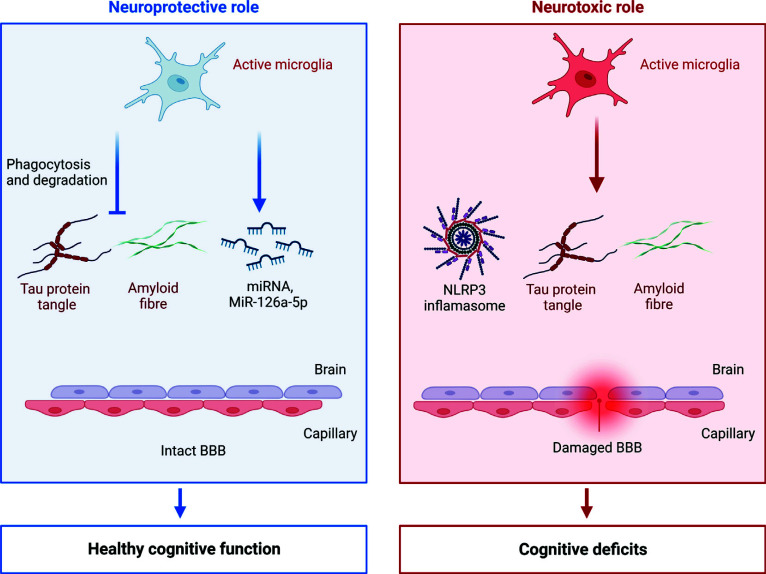
Dual effect of microglia on the BBB integrity. Microglia protect the BBB through phagocytosis and clearance of tau proteins and amyloid fibers, and *via* secretion of miRNA-126a-5p, which is beneficial for healthy cognitive function. However, microglia can also impair the BBB through NLRP3 inflammasome-mediated neuroinflammation, the production of tau proteins, and amyloid fibers, which contribute to cognitive deficits.

Microglia play a neuroprotective role at the beginning of CNS disorders. However, in addition to neuroprotective effects, microglia also have the potential to damage the BBB (Fig. **3**). Microglia are phagocytes that clear tissue debris, damaged cells, and other unneeded substances. This phagocytic activity means that microglia are involved in protecting BBB integrity. A recent study has illustrated the dual effects of microglia on the BBB. Under inflammatory stimulation, microglia are driven to cerebral vessels and accumulate, thereby increasing the permeability of the BBB. But simultaneously, the ablation of microglia increases the BBB permeability early during LPS-induced inflammation. The permeability further increases with the progression of the inflammation as the microglial protective effect is reversed [62].

The accumulation of the extracellular amyloid-β (Aβ) peptides induces microglial activation and the subsequent cytokine secretion. Aβ deposition is one of the characteristics of AD and results from the cleavage of amyloid precursor protein (APP) [63]. Cytokines, such as IL-1β and IL-33, can mediate the microglial activation and Aβ clearance [64]. TREM2 is one of the key receptors of microglial phagocytosis. It was reported that TREM2 knock-out mice have less Aβ clearing ability than wild-type mice, and that phagocytosis can be stimulated by mAb11 in a concentration-dependent manner. Mab11 works as an anti-Aβ antibody with amyloid binding properties and can initiate both TREM2-dependent and TREM2-independent engulfment of Aβ [65]. M2-like protective activation of microglia is reported to be transient but will switch to M1-like pathological activation if the resolution fails. The chronically activated microglia will consistently secrete pro-inflammatory mediators, which contribute to neurodegenerative diseases, including AD [66]. Microglial chronic activation may result in phagocytosis of endothelial cells or astrocytic endfeet [62, 67], leading to BBB damage and the loss of synapses [68]. Furthermore, the damage to the BBB is related to the neurotoxic Aβ deposition [69]. Aβ destroys TJ proteins and brain microvascular endothelial cells (BMEC) [69]. NLRP3 inflammasome-mediated neuroinflammation promotes the progression of AD. Microglia secrete adapter protein apoptosis-associated speck-like protein containing a CARD (ASC), one of the components of the NLRP3 inflammasome [70]. Therefore, NLPR3 may be a novel target for AD treatment.

#### 3.3.2 Microglia-endothelial Cells Interactions Resulting in BBB Disruption

**Fig. (4):**
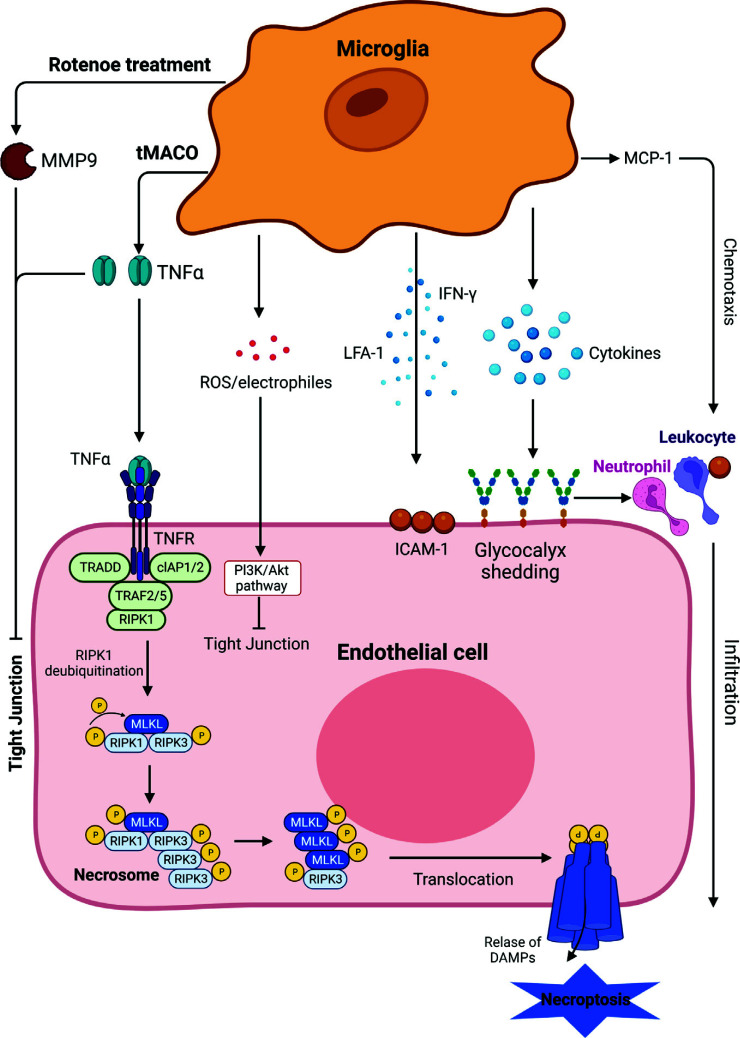
The effects of microglia on endothelial cells. Microglia may affect endothelial cells in different ways. Rotenone-treated microglia impair the TJs through secretion of MMP-9, and microglia-secreted TNF-α induces endothelial cell necroptosis in transient middle cerebral artery occlusion (tMCAO) mouse model. Stressed microglia-mediated ROS/electrophiles induce TJs impairment through the PI3K/Akt pathway in endothelial cells. Microglia-mediated cytokines may induce peripheral immune cells’ infiltration through upregulation of ICAM-1 and glycocalyx shedding on the cell membrane. Microglia-produced MCP-1 chemotaxis aggravates neutrophil and leukocyte infiltration into the brain. All these effects eventually result in BBB damage.

Endothelial cells restrict the entry of soluble substances and cells from the blood across the BBB into the brain. Microglia are part of the NVU, and they can release several pro-inflammatory cytokines, which can interact with the BBB (Fig. **4**). Necroptosis is a form of programmed necrotic cell death. A study evaluated endothelium necroptosis by detecting the expression levels of p-RIP1/RIP1 and p-MLKL/MLKL, which are hallmarks of necroptosis activation. TNF-α is the major trigger of necroptosis and apoptosis. When it binds to the TNF receptor on the cell membrane, receptor-interacting protein kinase 1 (RIP1) can be activated and further mixed lineage kinase domain-like proteins (MLKL) are assembled and phosphorylated. Decreasing secretion of TNF-α in microglia reduced p-RIP1/RIP1 and p-MLKL/MLKL after transient middle cerebral artery occlusion (Fig. **4**). This suggested that M1-like microglia are responsible for endothelial necroptosis [71]. Trans-endothelial electrical resistance (TEER) is a reliable and sensitive indicator of permeability and reflective of BBB integrity. TNF-α can decrease TEER of the BBB and attenuate the expression of occludins, ZO, and Cldn5 through the NF-κB signaling pathway or phosphatidylinositol-3 kinase (PI3K) [72].

MMPs are zinc-dependent endopeptidases family that can activate cytokines and chemokines. MMP-9 is a significant regulator of neuroinflammation, and its expression correlates with BBB dysfunction. A recent study suggested that when MMP9 is activated and expressed at a high level, it is associated with BBB damage and reduces TJ proteins in rotenone-treated mice. Rotenone, a microtubule assembly inhibitor and an inhibitor of the electron transport chain, decreases the expression of TJ proteins in a concentration-dependent manner in the substantia nigra [73]. The depletion of microglia using a CSF1R inhibitor and a protein synthesis inhibitor, PLX3397 and minocycline, decreased the activation of MMP9, which alleviated BBB dysfunction [73].

The EG covers the endothelial surface and is a key regulator of BBB functions [74]. One of the EG components is heparan sulfate (HS), which regulates the concentration of some cytokines. As a result, EG can buffer the pro-inflammatory cytokines [75]. The integrity of EG is significant, whereas it is a fragile structure that is easily damaged by various inflammatory and ischemic injuries, leading to endothelial dysfunctions. Depletion of EG leads to the adhesion of monocytes and the infiltration of macrophages [76]. Pro-inflammatory cytokines can destroy the EG, which may release VCAM and ICAM on the endothelial cells (Fig. **4**). The increase in adhesion and infiltration further exacerbates the inflammatory responses [77].

MECA-32 is a single-pass type II membrane homodimer glycoprotein in endothelial cells. It is a marker of CNS blood vessels with compromised vascular integrity. Depletion of microglia following chronic mild hypoxia increases MECA-32, suggesting that microglia may contribute to endothelial cells that are damaged through the reduction of MECA-32. Microglia can be activated and recruited to the cerebral blood vessels forming perivascular clusters and phagocytose endothelial cells, which results in the disintegration of blood vessels and further damages the BBB under inflammatory conditions [67].

Microglia respond to inflammation and CNS damage *via* phagocytosis and secretion of pro-inflammatory cytokines. Microglia can be activated by IFN-γ. Following injection of IFN-γ, microglial activation markers are increased at multiple timepoints, and the mRNA of several pro-inflammatory cytokines also significantly increases [78]. IL-12 and IL-18 can induce the mRNA expression of INF-γ in microglia, which further promotes the expression of major histocompatibility complex class II mRNA [79]. Cytokines stimulate adhesion molecules on brain microvascular endothelial cells, such as ICAM-1, which bind leukocyte ligands and transport active leukocytes into the CNS. In turn, this leads to the development of CNS disorders [80, 81]. In the Parkinson's disease, reactive microglia express lymphocyte function-associated antigen 1 (LFA-1), which has a critical role in inflammation. LFA-1-positive microglia aggregate and co-localize to ICAM-1-positive areas (Fig. **4**) [82]. ICAM-1 is also localized to phagocytic microglia [83]. This demonstrates the association between microglia and ICAM-1, and suggests that they are involved in the development of CNS disorders.

ROS are a regulator of pro-inflammatory cytokines and pro-inflammatory processes *via* different signaling pathways [84]. It is believed that ROS are secreted by NADPH oxidase (NOX) and that they are involved in immune responses. ROS damage BBB integrity through transient activation of the PI3K/AKT pathway *via* their upstream mediator, Rho, which disrupts the TJs [85]. Rho family GTPase is required for ROS-induced phosphorylation of protein kinase B (PKB). The actin cytoskeleton also compromises the integrity of the BBB and is associated with TJs [86]. Using a Rho-inhibitor can prevent the destruction of TJs and polymerization of the actin cytoskeleton [87].

Microglia and monocyte response to acute brain injury is regulated by monocyte chemoattractant protein (MCP)-1, the “CC” chemokine [88]. MCP-1 increases after lesions, as does its receptor, C-C motif chemokine receptor (CCR) 2. Accumulating evidence suggests that microglia, astrocytes, and BMEC express MCP-1 and CCR2 [89]. MCP-1 is also involved in AD, where it is found in primitive plaques, classic plaques, and reactive microglia [90]. Furthermore, MCP-1 induces neutrophil and leukocyte-secreting pro-inflammatory cytokines, such as IL-1β, which can damage BBB integrity [91]. IL-1β was found to be decreased in MCP-1-deficient mice. In addition to CCR2, the function of MCP-1 may also be dependent on the activity of plasmin, which is the activator of MCP-1 [92]. As for the destruction of the BBB, MCP-1 can activate PKC, leading to the removal of TJ proteins from the cell-cell border and reorganizing the actin cytoskeleton in BMECs (Fig. **4**) [93].

#### 3.3.3 Activated Microglia Trigger Alternations of Astrocyte Functions

**Fig. (5):**
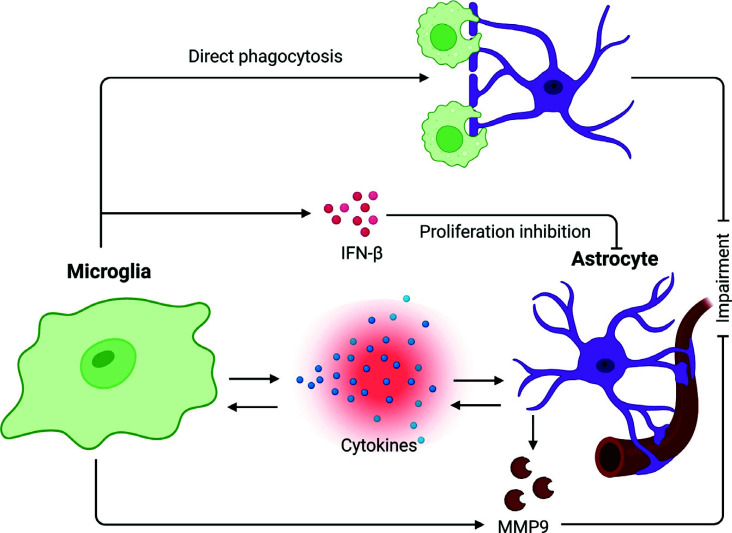
Schematic presentation of the effects of microglia on astrocytes. Microglia could communicate with astrocytes *via* cytokines, which may result in MMP-9 secretion and impair the BBB. Microglia could also secrete IFN-β to inhibit the proliferation of astrocytes or directly phagocytose the astrocyte endfeet, resulting in BBB damage.

Astrocytes restrict peripheral immune cells from crossing the BBB. The crosstalk between microglia and astrocytes has a critical role in responding to neuroinflammation. LPS-induced M1 microglia can release several pro-inflammatory cytokines, including IL-1 and TNF, which induce reactive astrocytes (A1) and damage the astrocytic capacity for metabolizing glutamate [10]. IL-1β induces a large number of astrocytes and increases the expression of MMP-9, which leads to BBB injury (Fig. **5**) [94]. Highly enriched astrocytes will release a small amount of pro-inflammatory factors in response to LPS stimulation but cannot be activated without microglia [95].

Microglia are activated through TLR4 to release pro-inflammatory cytokines, and astrocytes are reactive to TLR2, TLR3, and TLR4 after ICH [96]. Microglial and astrocyte phenotypes are altered by environmental factors. M2 microglia release IL-10, and A2 astrocytes express IL-10 receptor, which is related to neuronal repair [53]. Microglia mediated the neuroinflammatory activation of astrocytes when they were co-cultured. After preprocessing with LPS and using an NF-κB inhibitor, microglial expression of pro-inflammatory cytokines was found to be reduced, and consequently, cytokine release by astrocytes decreased [97]. Moreover, we have demonstrated that microglial IFN-β contributes to the inhibition of astrocyte proliferation, resulting in brain atrophy in a hypoxia-ischemic mouse model (Fig. **5**) [98].

Microglia phagocytosis also affects astrocytes. A research group analyzed microglia in the amygdala. ALDH1L1, an astrocytic marker, was found in the phagocytic cup of microglia, which suggests that microglia phagocytose astrocytic material [99]. Another recent study has reported that microglia express the phagocytic marker CD68 in MRL/lpr mice *via* gene analysis. Phagocytosis’ intensity was found to be positively correlated with the activation status of microglia. Using AQP4-positive puncta as a marker for possible astrocyte endfeet phagocytosis, immunoreactivity of AQP4 was observed in microglia phagosomes during inflammatory states, which suggests that microglia can directly phagocytose the astrocytic endfeet during LPS-induced inflammation [62]. Astrocyte endfeet are considered the key to the formation and maintenance of the BBB because many factors that astrocytes secrete can regulate the barrier function through ECs and TJs. The loss of astrocyte-vascular connections leads to disruption of the BBB in multiple sclerosis [100]. Therefore, microglial phagocytosis is a crucial response to inflammation, and such interaction with astrocytes causes damage to the BBB (Fig. **5**).

## 4 THE IMPACT OF BBB-RELEASED MEDIATORS ON MICROGLIAL ACTIVATION

Orosomucoid-2 (ORM2) and lipocalin 2 (LCN2) belong to the family of lipocalin proteins and are associated with microglial activation [101, 102]. Astrocytic ORM2 can bind to microglial CCR5, preventing the chemokine C-X-C motif ligand (CXCL)-4-CCR5 interaction, which activates microglia. ORM2 downregulates the activation of microglia and promotes an anti-inflammatory response [103]. Additionally, glial cell line-derived neurotrophic factor (GDNF), cerebral dopamine neurotrophic factor (CDNF), and brain-derived neurotrophic factor (BDNF) are secreted by astrocytes, and are involved in microglial regulation [104-106]. Astrocyte-derived MCP-1/CCL2 and IFN-γ inducible protein (IP)-10/CXCL10 are responsible for microglial activation as well as motility and subsequent neurodegeneration in inflammatory demyelinating diseases of the CNS [107].

Astrocytes also regulate microglial migration and phagocytosis. Plasminogen activator inhibitor type 1 (PAI-1) is mainly produced by astrocytes and is an important mediator of microglia-astrocyte crosstalk. PAI-1 promotes microglial migration *in vivo* and *in vitro via* the low-density lipoprotein receptor-related protein (LRP)-1/Janus kinase (JAK)/STAT1 axis under inflammatory conditions [108]. Astrocytes can regulate ROS production to manipulate the microglial activation in HIV-mediated neuropathogenesis. HIV transactivating regulatory protein (Tat)-stimulated astrocytes release miR-9, which can be recognized by microglia and promote microglial migration [109]. In AD models, complement factor C3 expression can be induced in astrocytes by Aβ, which interacts with the microglial C3a receptor (C3aR) to regulate microglial phagocytosis [110]. Therefore, controlling astrocytic C3 may regulate the microglial clearance of Aβ (Table **2**).

Endothelial cells produce cytokines and chemokines, as do pericytes, which activate microglia [111]. Pericytes release MMP-9 and inflammatory molecules, including IL-1β, IL-6, TNF-α, and IFN-γ, when exposed to the cytokines [111, 112]. They promote the transformation of microglia and astrocytes into pro-inflammatory phenotypes. IL-6 mediates neurovascular dysfunction in neurodegenerative diseases, and it can amplify the TNF-induced iNOS mRNA expression in microglia [113]. Furthermore, pericytes not only secrete anti-inflammatory molecules, like IL-33 and C-X3-C motif chemokine ligand 1 (CX3CL1), to make microglia exhibit anti-inflammatory phenotype, but also promote the release of pro-inflammatory cytokines, leading to the breakdown of the BBB *in vitro* and inducing the activation of pro-inflammatory state of microglia, astrocytes, and endothelial cells [114-116].

CX3CL1 contributes to attenuating neuroinflammation, and its receptor is only expressed in microglia. CX3CL1 is involved in the interaction of neurons and microglia, and CX3CL1-CX3CR1 damage dysregulates microglial responses and leads to neuronal injury [117]. The interaction and further signaling of CX3CL1-CX3CR1 can maintain microglial resting phenotype and inhibit the secretion of TNF-α, NO, and superoxide.

CX3CL1 also regulates other pro-inflammatory molecules. IL-1β, IL-6, and macrophage scavenger receptor 1 (MSR1) expression increase in the membrane-anchored CX3CL1-deficient mice, which is related to the activation of microglial p38 mitogen-activated protein kinase (MAPK) and response to Aβ phagocytosis mediated by MSR1 [118]. Additionally, CX3CL1 plays different roles in different pathologies, and it is not necessarily always protective, but also promotes activation and the release of pro-inflammatory factors [119]. During HIV-associated dementia, overexpressing CX3CL1 recruits and stimulates HIV-infected mononuclear phagocytes, including microglia, to release toxic factors. Endothelial cell-produced CX3CL1 can interact with CX3CR1 on natural killer (NK) cells, and activated NK cells may reversely lead to endothelium damage. This suggests that CX3CL1 has a dual role in the CNS, which depends on the activated phenotype of microglia [120].

Several pro-inflammatory cytokines increase in the striatum of patients with Huntington’s disease (HD). Microglia are involved in the progression of the disease, and their number increase in the striatum [121]. MMP-3 activation is increased in HD and can modify huntingtin proteolysis and toxicity [122]. Furthermore, MMP-3 induces the microglial release of pro-inflammatory cytokines [123]. A previous study that used TNF-α as a marker to quantify cytokines has observed MMP-3 to induce an increase of TNF-α in microglia. Moreover, the induction of microglial activity and the release of pro-inflammatory cytokines depend on extracellular signal-regulated kinase (ERK) and NF-κB signaling pathway [123].

Vascular endothelial growth factor (VEGF) is highly specific and is related to the stimulation of the endothelial proliferation, migration, and formation of vessels. The fms-like tyrosine kinase-1 (Flt-1) subtype of the VEGF receptor can mediate the chemotaxis of reactive immune cells, including microglia, and has an important role in AD [124]. VEGF has also been implicated in stroke, during which the level of VEGF increases in astrocytes and endothelial cells [125]. Extracellular vesicles detach from astrocytes carrying VEGF. Ischemia/reperfusion during acute stroke damages the BBB, activates glial cells, and induces pericytes apoptosis. VEGF is initially increased and normalized. Then, this process repeats [126]. The subtypes of VEGF regulate microglia. For example, VEGF-C can promote the M2 phenotype in a VEGFR-dependent manner, and VEGF can inhibit microglia from releasing pro-inflammatory cytokines [127].

## CONCLUSION

Microglia are the key immune cells that act as the initial line of defense in the CNS. They immediately respond to the pathogen invasion by releasing cytokines to mediate an inflammatory response. Activated microglia can differentiate into two phenotypes and release various cytokines that are either neuroprotective or neurotoxic. Different cytokines have different roles in CNS disorders. Some can further damage the neurons and the BBB, whereas others enhance phagocytic function to speed up the clearance of necrotic tissue and the repair of damaged tissue [128]. The BBB is important for CNS homeostasis. High BBB permeability is associated with many neurological diseases, which are also closely associated with microglia. The BBB is composed of several cell types that can also release cytokines to regulate the activation or quiescence of microglia and the progression of neurological diseases.

We have discussed the communication between microglia and the BBB, including their characteristics, and highlighted their roles in mediating or attenuating the neuroinflammatory progression. Microglial activation may be one of the earliest phenomena involved in the progression of aging and neurodegeneration. The role of microglia relating to BBB integrity is essential for our understanding of BBB dysfunction in CNS diseases. At present, many studies have focused on several specific cytokines mediating the interactions, such as TNF-α, CX3CL1, and MMP-3. There are still many unexplored mechanisms and functions of the other mediators that may be useful for the development of novel therapies for neurological diseases.

## Figures and Tables

**Table 1:** Factors of microglial polarization.

**Factors**	**Type of Factors**	**Major Activities**	**References**
IFN-γ	Pro-inflammatory factor	Released by M1 microglia	[48]
TNF-α	Pro-inflammatory factor	Released by M1 microglia and induces neuronal death and apoptosis	[50]
IL-1β	Pro-inflammatory factor	Released by M1 microglia and induces neuronal death and apoptosis	[50]
NO	Pro-inflammatory factor	Released by M1 microglia	[48]
ROS	Pro-inflammatory factor	Released by M1 microglia	[48]
iNOS	Pro-inflammatory factor	Released by M1 microglia	[48]
IL-6	Pro-inflammatory factor	Released by M1 microglia and reduces microglial process length, the number of branching points and terminal points	[49]
IL-10	Anti-inflammatory factor	Converts microglial phenotype from M1 to M2	[53]
TGF-β	Anti-inflammatory factor	Converts microglial phenotype from M1 to M2	[53]
IL-4	Anti-inflammatory factor	Promotes differentiation to M2 phenotype and reduces neuronal apoptosis	[54]

**Table 2:** Factors from the BBB and their effects on microglia.

**Factors**	**Cell Source**	**Effects on Microglia**	**References**
ORM-2	Astrocyte	Deregulates microglial activation and migration	[101]
LCN2	Astrocyte	Activates microglial M1 phenotype	[102]
GDNF	Astrocyte	Inhibits activation of microglia	[104]
CDNF	Astrocyte	Promotes polarization to anti-inflammatory microglia phenotype	[105]
BDNF	Astrocyte	Promotes microglial activation	[106]
(MCP)-1/CCL2	Astrocyte	Promotes microglial activation and migration	[97]
(IP)-10/CXCL10	Astrocyte	Promotes microglial activation and migration	[97]
PAI-1	Astrocyte	Promotes microglial migration	[108]
miR-9	Astrocyte	Promotes microglial migration	[109]
Complement C3	Astrocyte	Regulates microglial phagocytosis	[110]
IL-1β	Pericyte	Promotes microglial activation	[111]
IL-6	Pericyte	Promotes microglial activation	[111]
IFN-γ	Pericyte	Promotes microglial activation	[111]
MMP-9	Pericyte	Promotes microglial activation	[111]
TNFα	Pericyte	Promotes microglial activation	[112]
CX3CL1	Pericyte	Promotes microglial anti-inflammatory phenotype or inflammatory phenotype	[114, 120]
IL-33	Pericyte	Promotes microglial recruitment, phagocytosis, and polarization to anti-inflammatory phenotype	[115, 116]
MMP-3	Pericyte	Promotes microglial activation	[123]
VEGF	Astrocyte, endothelial cell	Activates microglial M2 phenotype or inhibits microglia-releasing pro-inflammatory factors	[127]

## LIST OF ABBREVIATIONS

Aβ: Amyloid-β
AD: Alzheimer’s Disease
AJ: Adherens Junction
APP: Amyloid Precursor Protein
AQP4: Aquaporin 4
ASC: Apoptosis-associated Speck-like Protein Containing a CARD
BBB: Blood-brain Barrier
BDNF: Brain-derived Neurotrophic Factor
BMEC: Brain Microvascular Endothelial Cell
C3aR: C3a Receptor
C4b: Complement Fragment 4b
CCL2: (C-C motif) Ligand 2
CCR: C-C motif Chemokine Receptor
CDNF: Cerebral Dopamine Neurotrophic Factor
Cldn5: Claudin-5
CNS: Central Nervous System
COX: Cyclooxygenase
CSF1R: Colony Stimulating Factor 1 Receptor
CX3CL1: C-X3-C Motif Chemokine Ligand 1
CXCL8: C-X-C Chemokine Ligand 8
CX3CR1: Fractalkine Receptor
DAM: Disease-associated Microglia
ECM: Extracellular Matrix
EG: Endothelial Glycocalyx
ERK: Extracellular Signal-regulated Kinase
Flt-1: Fms-like Tyrosine Kinase-1
GDNF: Glial Cell Line-derived Neurotrophic Factor
HD: Huntington’s Disease
HS: Heparan Sulfate
ICAM: Intercellular Adhesion Molecule
ICH: Intracerebral Hemorrhage
IFN: Interferon
IL: Interleukin
iNOS: Inducible Nitric Oxide Synthase
LCN2: Lipocalin 2
LFA-1: Lymphocyte Function-associated Antigen 1
LPS: Lipopolysaccharide
LRP: Lipoprotein Receptor-related Protein
MAPK: Mitogen-activated Protein Kinase
MCP: Monocyte Chemoattractant Protein
M-CSF-1R: Macrophage Colony-stimulating Factor-1 Receptor
MLKL: Mixed Lineage Kinase Domain-like Protein
MMP: Matrix Metalloproteinase
MSR1: Macrophage Scavenger Receptor 1
NK: Natural Killer
NO: Nitric Oxide
NOS: Nitric Oxide Synthase
NOX: NADPH Oxidase
NVU: Neurovascular Unit
ORM2: Orosomucoid-2
PAI-1: Plasminogen Activator Inhibitor Type 1
Pcsk1n: Proprotein Convertase Subtilisin/Kexin Type 1 Inhibitor
PI3K: Phosphatidylinositol-3 Kinase
PKB: Protein Kinase B
PKC: Protein Kinase C
Ppp1r14a: Protein Phosphatase 1 Regulatory Inhibitor Subunit 14A
RhoK: Rho Kinase
RIP1: Receptor-interacting Protein Kinase 1
ROS: Reactive Oxygen Species
TEER: Trans-endothelial Electrical Resistance
TGF-α: Transforming Growth Factor-α
TJ: Tight Junction
TLR: Toll-like Receptor
TNF-α: Tumor Necrosis Factor-α
TREM2: Triggering Receptors Expressed on Myeloid Cells 2
vBBB: Vascular Blood-brain Barrier
VCAM: Vascular Cell Adhesion Molecule
VEGF: Vascular Endothelial Growth Factor
ZO: Zonula Occludens
